# Toxicity of λ-cyhalothrin and fenpyroximate on *Nannotrigona testaceicornis* - dataset

**DOI:** 10.1016/j.dib.2026.112714

**Published:** 2026-03-20

**Authors:** Nuno G.C. Ferreira, Jaíne Santos Rebouças Vieira, Jefferson Alves dos Santos, Maiara Janine Machado Caldas, Maria Angélica Pereira de Carvalho Costa, Ana Tereza Bittencourt Guimarães, Carlos Alfredo Lopes de Carvalho

**Affiliations:** a CIIMAR/CIMAR LA, Centro Interdisciplinar de Investigação Marinha e Ambiental, Universidade do Porto, Terminal de Cruzeiros do Porto de Leixões, 4450-208 Matosinhos, Portugal; b School of Biosciences – Cardiff University. Museum Avenue, Cardiff CF10 3AX, Wales, UK; c Grupo de Pesquisa Insecta, Universidade Federal do Recôncavo da Bahia, Cruz das Almas, Bahia, Brazil; d Ecotoxicology and Landscape Research Group, Rua Universitária n. 2069, Cascavel-PR, Brazil; e Pós-graduação em Biociências e Saúde, Universidade Estadual do Oeste do Paraná. Rua Universitária n. 2069, Cascavel-PR, Brazil

**Keywords:** Pollinators, Meliponini, Ecotoxicology, λ-Cyhalothrin, Fenpyroximate

## Abstract

The use of pesticides in agriculture boosts food production yields. However, pesticides are directly linked to declining pollinator populations, consequently impacting crop productivity. Assessing the effects of pesticides on pollinating bees is crucial for informing crop management strategies and public policies. This manuscript provides a dataset from the study that evaluated the toxicity of the active ingredients λ-cyhalothrin (insecticide) and fenpyroximate (acaricide) on the stingless bee species *Nannotrigona testaceicornis* (Apidae: Meliponini). The results include measurements of bee's weight, survival, ingestion rates, behavioural responses, and the bee behavioural stress index (BSI). This dataset can be further used for ecological risk assessments, determination of risk factors and quotients, and developing behavioural indices, models, and clustering analyses. Policymakers can use this dataset to support their decision-making processes.

Specifications Table

**Table:** 

Subject	Environmental Sciences and Biological Sciences: Behaviour, Management, Monitoring, Policy and Law
Specific subject area	Environmental Toxicology
Type of data	Tables (.xlsx format) Raw and simplified variables created by consolidating raw data.
Data collection	The dataset contains two files, each representing three exposure routes for the insecticide λ-cyhalothrin and the acaricide fenpyroximate. The weight variables were collected using Shimadzu scale (Model: AUY220). Live and dead animals were counted. Food consumption rates were measured based on the weight of food offered and remaining at different sampling times. Behaviour was recorded by counting the number of organisms: agitated, disoriented, paralysed, prostrated, with reduced coordination, wing fluttering and self-cleaning. For the contact surface exposure, chewing on paper was also recorded. These parameters were used to calculate the bee behavioural stress index (BSI).
Data source location	Data was collected within the laboratory of the "Grupo de Pesquisa Insecta" - Universidade Federal do Recôncavo da Bahia, Cruz das Almas, Bahia, Brazil.
Data accessibility	Data is available within this manuscript and also at Repository name: ZENODO Data identification number: DOI: /10.5281/zenodo.14359028 Direct URL to data: https://doi.org/10.5281/zenodo.14359028
Related research article	Rebouças, J.S.; dos Santos, J.A.; Caldas, M.J.M.; Costa, M.A.P.C.; Guimarães, A.T.B.; Ferreira, N.G.C.; de Carvalho, C.A.L. Toxicity of λ-cyhalothrin and fenpyroximate on Nannotrigona testaceicornis. Environmental Science and Pollution Research https://doi.org/10.1007/s11356–025–37169–7

## 1 Value of the Data

• This dataset offers raw and analysed bee toxicity data that can be integrated with future datasets from other bee species or chemicals generated by other researchers. It can be used to calculate toxicity parameters and compare the effectiveness of the tested compounds with those of other compounds.
• The data presented allows readers to understand how different classes of pesticides interact with bee species.
• The bee toxicity data can help researchers develop intra- and interspecific models to assess insecticide and fungicide toxicity.
• Ecological risk assessors and policymakers can use this data to better understand and account for variability in how different species respond to contaminants and for developing conservation policies.
• Researchers and regulatory agencies can also use this data to develop new toxicity testing methodologies based on behavioural tests that align with current legal requirements or complement existing standard guidelines.

## 2 Background

Agriculture is a critical global activity, covering about 37.7% of the world's land and playing a vital role in food production and employment [1]. However, the growing demand for food has led to increased pesticide use [2], causing significant damage to biodiversity, particularly pollinators like stingless bees (Meliponini) [3]. Pesticide exposure can have devastating effects on bees, leading to immediate mortality or sublethal impacts [4]. Current OECD guidelines may not fully capture bees' complex behavioural responses to these chemicals.

In Brazil, >251 species of stingless bees exist [5], of which *Nannotrigona testaceicornis* is a crucial pollinator for various economically important crops like mango and melon. These play a crucial role in the exportation of Brazil's major fruit-producing region, the San Francisco Valley. Still, large-scale production often involves conventional pest control methods using pesticides like λ-cyhalothrin and fenpyroximate, commonly used in the area [6].

This study evaluated the toxicity of λ-cyhalothrin and fenpyroximate on *N. testaceicornis* through ingestion, topical application, and surface contact at levels recommended for pest control in mango and melon crops. It introduces a new bee behavioural stress index (BSI) to provide a more comprehensive assessment of bee health and identify subtle sublethal effects.

## 3 Data Description

The presented dataset includes two files as follows:

**File 1 - Fenpyroximate.xlsx –** contains the raw and processed toxicity data for the acaricidefenpyroximate. It has eight sheets for the ingestion, topical and surface contact exposure routes as follows:

• **Mortality_FEN_ingestion –** presents the raw mortality data observed for the fenpyroximate exposure through the ingestion route;
• **Behaviour_FEN_Ingestion –** presents the behavioural changes observed for the fenpyroximate exposure through the ingestion route;
• **Consumption_FEN_Ingestion –** presents the food consumption rates for the fenpyroximate exposure per sampling time, total consumption rate from the beginning of the exposure until the specific sampling time, the food consumption rates per sampling time per bee, total consumption rate from the beginning of the exposure until the specific sampling time per bee;
• **Mortality_FEN_Topical -** presents the raw mortality data observed for the fenpyroximate exposure through the topical route;
• **Behaviour_FEN_Topical –** presents the behavioural changes observed for the fenpyroximate exposure through the topical route;
• **Mortality_FEN_Contact_Surface -** presents the raw mortality data observed for the fenpyroximate exposure through the contact surface route;
• **Behaviour_FEN_Contact_Surface –** presents the behavioural changes observed for the fenpyroximate exposure through the contact surface route;
• **BSI_FEN -** presents the calculated Bee Behavioural Stress Index (BSI) for the exposure to fenpyroximate to the three exposure routes.

**File 2 – Lambda_Cyhalothrin.xlsx –** contains the raw and processed toxicity data for the insecticide λ-cyhalothrin. It has eight sheets for the ingestion, topical and surface contact exposure routes as follows:

• **Mortality_LC_ingestion –** presents the raw mortality data observed for the exposure of λ-cyhalothrin through the ingestion route;
• **Behaviour_LC_Ingestion –** presents the behavioural changes observed for the λ-cyhalothrin exposure through the ingestion route;
• **Consumption_LC_Ingestion –** presents the food consumption rates for the λ-cyhalothrin exposure per sampling time, total consumption rate from the beginning of the exposure until the specific sampling time, the food consumption rates per sampling time per bee, total consumption rate from the beginning of the exposure until the specific sampling time per bee;
• **Mortality_LC_Topical -** presents the raw mortality data observed for the λ-cyhalothrin exposure through the topical route;
• **Behaviour_LC_Topical –** presents the behavioural changes observed for the λ-cyhalothrin exposure through the topical route;
• **Mortality_LC_Contact_Surface -** presents the raw mortality data observed for the λ-cyhalothrin exposure through the contact surface route;
• **Behaviour_LC_ Contact_Surface –** presents the behavioural changes observed for the λ-cyhalothrin exposure through the contact surface route;
• **BSI_LC –** presents the calculated Bee Behavioural Stress Index (BSI) for the exposure of λ-cyhalothrin to the three exposure routes.

## 4 Experimental Design, Materials and Methods

The methodology is described in detail in the related research article by Rebouças et al. (2025) [7]. This section provides a concise overview of the methodology.

### 4.1 Bee sampling and experimental design

Worker bees of *Nannotrigona testaceicornis* (*n* = 1,173) were obtained from 16 standardised colonies maintained at the Cruz das Almas meliponary in Bahia, Brazil. Sampling was conducted during periods of maximum foraging activity by sealing colony entrances with wax and capturing returning foragers using 500 mL polypropylene collection vessels.

Following collection, individual bees were observed to identify potential abnormalities. Any organisms exhibiting atypical behaviour were excluded from the study and substituted with behaviourally normal organisms prior to pesticide exposure. Afterwards, bees were immobilised through controlled cold anaesthesia (−14 °*C* ± 2 °C for 1 min 25 *sec*) before being transferred to modified 500 mL experimental cages [8]. Each experimental treatment comprised three independent replicates (cages), with each replicate containing 10 individual bees. Throughout the exposure period, experimental units were maintained in controlled environment chambers following established protocols adapted from Botina et al. (2020) [7] and OECD guidelines #213 [9] and #214 [10], with environmental conditions set at 28 ± 2 °C and 70 ± 5% relative humidity to accommodate species-specific physiological requirements.

### 4.2 Concentrations and solution preparation

Bees were exposed to a range of concentraitons of λ-cyhalothrin (CAS No 91,465-08–6) and fenpyroximate (CAS No 134,098-61–6). The experimental concentrations for λ-cyhalothrin and fenpyroximate were selected based on the lowest and highest doses recommended for field application were obtained from Agrofit (https://agrofit.agricultura.gov.br/) [6]. The estimated LC_50_ values were obtained through preliminary tests with social bee species [11] or from the study of Pilling and Jepson (1993) [12] as follows:

### 4.3 λ-cyhalothrin concentrations

A. Ingestion (μg/mL)

• 1.440 ½ LC_50_ *Melipona quadrifasciata anthidioides*[11]
• 2.880 LC_50_ *Melipona quadrifasciata anthidioides* [11]
• 5.760 2x LC_50_ *Melipona quadrifasciata anthidioides* [11]
• 10.000 Lower RAFD (mango cultures) [6]
• 83.300 Higher RAFD (mango cultures) [6]

B. Topical (μg/μL)

• 0.010 Lower RAFD (mango cultures) [6]
• 0.083 Higher RAFD (melon cultures) [6]
• 0.345 5 × LC_50_ *Apis mellifera* [12]
• 0.690 10 × LC_50_ *Apis mellifera* [12]
• 1.380 20 × LC_50_ *Apis mellifera* [12]

C. Contact surface (μg/mL)

• 41.650 ½ Higher RAFD (melon cultures) [6]
• 83.300 Higher RAFD (melon cultures) [6]
• 124.950 1.5 × Higher RAFD (melon cultures) [6]
• 166.600 2 × Higher RAFD (melon cultures) [6]

### 4.4 Fenpyroximate concentrations

A. Ingestion (μg/mL)

• 12.500 ¼ Higher RAFD (mango cultures) [6]
• 25.000 ½ Higher RAFD (mango cultures) [6]
• 37.500 Lower RAFD (mango cultures) [6]
• 50.000 Higher RAFD (mango cultures) [6]

B. Topical (μg/μL)

• 0.025 ½ Higher RAFD (mango cultures) [6]
• 0.037 Lower RAFD (mango cultures) [6]
• 0.050 Higher RAFD (mango cultures) [6]
• 0.100 2 × Higher RAFD (mango cultures) [6]

C. Contact surface (μg/mL)

• 18.870 ½ Lower RAFD (mango cultures) [6]
• 37.750 Lower RAFD (mango cultures) [6]
• 50.000 Higher RAFD (mango cultures) [6]
• 73.500 ∼1.5 × Higher RAFD (mango cultures) [6]
• 100.000 2 × Higher RAFD (mango cultures) [6]

Pesticide formulations were prepared through solvent dilution according to the exposure route administered. For ingestion exposures, λ-cyhalothrin was dissolved in absolute ethyl alcohol (99.5%) while fenpyroximate was dissolved in acetone (99.5%). The initial pesticide-solvent mixture was subsequently combined with a 1:1 sucrose solution (mass/volume ratio of sucrose to distilled water) to establish the stock solution at maximum intended concentration. Serial dilutions of this stock solution using sucrose solution as the diluent yielded the intermediate and lower exposure concentrations required for the ingestion route. Topical application and contact surface exposure routes employed a similar preparation protocol, with the exception that distilledwater replaced sucrose solution as the solvent. For λ-cyhalothrin, the solvent concentrations were 0.67% for ingestion, 20% for topical application, and 0.67% for contact surfaces. Fenpyroximate had solvent concentrations of 0.4% for ingestion, 20% for topical, and 0.5% for contact surfaces.

### 4.5 Bioassay via ingestion

Experimental cages housing the bee colonies were maintained under controlled dark conditions at 28 °*C* ± 2 °C and 70% ± 5% relative humidity for an acclimation period of two hours, during which food and water access were restricted. Following this initial period, each cage was provisioned with a sucrose solution (1:1 mass/volume ratio of sucrose to distilled water) formulated with the corresponding pesticide treatment. For the control treatments, sucrose solution or sucrose solution with the same proportion of solvent as the initial solution was used. To prevent accidental mortality through drowning during solution consumption, cotton wool was inserted into microtubes serving as the feeding apparatus. In addition to the treatment solutions, all experimental groups maintained continuous access to mineral water ad libitum throughout the exposure period.

At designated evaluation intervals, microtubes containing the treatment or control solutions were removed, weighed, and assessed for depletion. When necessary, microtubes were replenished using the identical procedure . Following each evaluation interval, the following quantitative parameters were calculated based on recorded food consumption and the number of surviving organisms at the previous time: total food consumption (g/cage), the average consumption per bee (g/bee), and the average amount of active ingredient consumed per bee in each treatment (µg/bee) [13].

### 4.6 Topical bioassay

Worker bees were initially immobilised through controlled hypothermia (−14 °*C* ± 2 °C for 1 min 25 *sec*) prior to treatment application. A volume of 1 μL of the designated pesticide treatment solution was subsequently applied topically to the dorsal surface of the thoracic region of each bee. Control organisms received equivalent volumes of distilledwater or solvent-matched solutions maintaining the same solvent proportion as the experimental treatment preparations [10]. Following topical application, bees were provided ad libitum access to drinking water and a nutritive sucrose solution (1:1 mass/volume ratio of sucrose to distilled water).

### 4.7 Bioassay via contact surface

A Limatec® spray tower [14] was employed to uniformly distribute 1 mL of the designated treatment solution onto the interior surfaces of experimental cages that had been lined with Whatman® No 1 filter paper [15]. The treated cages were subsequently allowed to air-dry for 30 min at ambient temperature to permit complete solvent evaporation and pesticide fixation to the filter paper substrate. Following the drying period, ten individual worker bees were introduced into each prepared cage. Within each cage, two 1.5 mL microtubes were positioned to provide essential resources: one microtube contained water-saturated cotton wool to serve as the hydration source, whilst the second microtube held a nutritive sucrose solution (1:1 mass/volume ratio of sucrose to distilled water).

### 4.8 Bees's behavioural stress index

Bee survival rates and the sublethal effects were systematically evaluated at designated time intervals: (1, 6, 12, 24, 48, 72, and 96 h) [15,16]. Mortality was determined using a standardised criterion: individual bees were classified as deceased if they exhibited no response to mechanical stimulation applied via gentle contact with a soft-bristled brush [17]. In addition sublethal physiological and behavioural alterations were recorded and categorised as the following manifestations: disorientation, paralysis, prostration, impaired locomotor activity, agitation, wing tremor, self-directed grooming, and paper-chewing behaviour (contact surface exposure route only) [13,15].

The bee behavioural stress index (BSI) was determined according to the standardised protocol outlined by Rebouças et al. (2025) [7].

### 4.9 Statistical analysis

All statistical analyses were conducted in R software at a significance level of α=0.05.

**Survival Analysis:** Kaplan-Meier survival curves were generated using the Survival package [18] to assess bee survivorship trajectories across treatment groups. Curve comparisons were performed using the Log-Rank test [19], with pairwise treatment comparisons conducted when significance was detected using the ``pairwise_survdiff'' function (survminer package)[20] and Benjamini-Hochberg correction.

**Ingestion Exposure:** Cumulative sucrose consumption per bee over the 96-hour evaluation period was calculated [13]. Data were screened for normality (Shapiro-Wilk test) and homogeneity of variance (Bartlett's test). Parametric data meeting these assumptions were analysed using one-way ANOVA ("dic" function, ExpDes.pt package) [21], followed by the Scott-Knott post-hoc test for mean comparison. Data points exceeding the outlier threshold (Q3 + 1.5 × interquartile range or Q1 − 1.5 × interquartile range) were excluded from consumption analysis.

**Dose-Response Analysis:** Median lethal concentration (LC_50_) and median lethal dose (LD_50_ and LD_50_wt-adjusted) were estimated using log-logistic regression ("drm" function, drc package) on 96-hour mortality data. Parameter estimates were derived using the ``ED'' function (drc package) [22].

**Behavioural Stress Index:** Linear mixed models were fitted with pesticide concentration (λ-cyhalothrin and fenpyroximate) as fixed effects across exposure routes (ingestion, topical, contact surface) over 96 h using the lme4 package [23]. Treatment comparisons were conducted using contrast analysis (emmeans package) [24].

## Limitations

Not applicable

## Ethics Statement

This experiment involved invertebrate organisms and is in line with the EU Directive 2010/63/EU for animal experiments. Permission to collect and perform the ecotoxicological exposures were obtained from the Biodiversity Authorisation and Information System (SISBIO) under licence number 84,168–1.

## CRediT Author Statement

**N.G.C.F.:** Conceptualisation, methodology, validation, formal analysis, investigation, resources, data curation, writing - original draft, visualisation, project administration, funding acquisition; **J.S.R.V.:** Formal analysis, investigation, visualisation; **J.A.S.:** Formal analysis, investigation; **M.J.M.C.:** Investigation; **M.A.P.C.C.:** Investigation; **A.T.B.G.:** Methodology, validation, formal analysis, investigation, data curation, writing - original draft, visualisation; **C.A.L.C.:** Conceptualisation, methodology, validation, formal analysis, investigation, resources, supervision. All authors contributed to the review and editing of the original manuscript.

## Data Availability

ZENODOLambda-cyhalothrin and fenpyroximate Datasert (Original data).
